# Room-temperature superionic-phase nanocrystals synthesized with a twinned lattice

**DOI:** 10.1038/s41467-019-11229-2

**Published:** 2019-07-23

**Authors:** Jianxiao Gong, Prashant K. Jain

**Affiliations:** 1 Department of Chemistry, University of Illinois at Urbana-Champaign, Urbana, IL 61801 USA; 2 Materials Research Laboratory, University of Illinois at Urbana-Champaign, Urbana, IL 61801 USA; 3 Beckman Institute of Advanced Science and Technology, University of Illinois at Urbana-Champaign, Urbana, IL 61801 USA; 4 Department of Physics, University of Illinois at Urbana-Champaign, Urbana, IL 61801 USA

**Keywords:** Nanoparticles, Materials chemistry, Synthesis and processing, Materials for energy and catalysis

## Abstract

The engineering of nanoscale features enables the properties of solid-state materials to be tuned. Here, we show the tunable preparation of cuprous sulfide nanocrystals ranging in internal structures from single-domain to multi-domain. The synthetic method utilizes in-situ oxidation to grow nanocrystals with a controlled degree of copper deficiency. Copper-deficient nanocrystals spontaneously undergo twinning to a multi-domain structure. Nanocrystals with twinned domains exhibit markedly altered crystallographic phase and phase transition characteristics as compared to single-domain nanocrystals. In the presence of twin boundaries, the temperature for transition from the ordered phase to the high-copper-mobility superionic phase is depressed. Whereas the superionic phase is stable in the bulk only above ca. 100 °C, cuprous sulfide nanocrystals of ca. 7 nm diameter and a twinned structure are stable in the superionic phase well below ambient temperature. These findings demonstrate twinning to be a structural handle for nanoscale materials design and enable applications for an earth-abundant mineral in solid electrolytes for Li-S batteries.

## Abstract (web summary)

The ability to control the internal domain structure of a nanocrystal represents a new direction in nanomaterials design. Here, the authors develop a method to controllably introduce twin boundaries in cuprous sulfide nanocrystals, and find that twinning stabilizes these nanocrystals in the superionic phase well below room temperature.

## Introduction

Crystal twinning, wherein two separate crystal domains are symmetrically joined via shared lattice positions, is commonly encountered in minerals and industrial materials^1^. Such twin defects influence the structural and mechanical properties of materials;^2–5^ for instance, shock hardening of stainless steel is thought to result from twin boundaries^2,3,6^. Twinning has also been found in metal and semiconductor nanocrystals (NCs)^2,4,5,7–11^, where it has been exploited for the synthesis of anisotropic nanostructures^11–13^, the exploration of structure-dependence of catalytic, optical and mechanical properties^2,6^, and the formation of ultrahard materials^3,8,11,13^. We encounter twinning in NCs of copper sulfide (Cu_2−*x*_S).

Cu_2−*x*_S is an ionic solid attracting broad interest due to its potential as a solid electrolyte for batteries. The ambient-temperature phase of Cu_2−*x*_S, called low chalcocite, has low Cu^+^ mobility^14^. Above 104 °C, bulk Cu_2−*x*_S transitions to a superionic phase, called high chalcocite, wherein the Cu^+^ form a highly mobile, liquid-like sub-lattice within a rigid, immobile hexagonally close-packed S^2−^ framework^14,15^. While such superionic phases are promising as solid electrolytes due to their high ion conductance (>10^−3^ S cm^−1^)^16^, the high temperatures required to access superionic phases pose an obstacle for their use in batteries.

Here, we demonstrate that NCs engineered with twinned domains exhibit a superionic phase at temperatures within the safe operating range of batteries. Specifically, we describe a method for the controllable introduction of twin boundaries in Cu_2−*x*_S NCs. We find that the twin boundaries thus introduced stabilize the high-temperature superionic form of Cu_2−*x*_S in the NCs. In contrast to the bulk, Cu_2−*x*_S NCs synthesized with twin boundaries exhibit the superionic phase at a dramatically reduced temperature (as low as 0 °C). In general, the findings show that crystal twinning can be employed as a structural handle for tuning ionic structure and transport on the nanoscale.

## Results and discussion

### Synthesis of Cu_2−*x*_S NCs with controlled Cu deficiency levels

Cu deficiency is a salient feature of Cu_2−*x*_S^17,18^. In near-stoichiometric Cu(II) sulfide, *x* can range from 0–0.07^14,19,20^. We synthesized NCs with a controlled Cu-deficiency level. To achieve this, we modified a published synthesis protocol to allow for in situ oxidation (see Methods)^21^. Specifically, a controlled volume of air was injected into the reaction flask. We found that the presence of air during synthesis resulted in the oxidative removal of Cu from the Cu_2_S lattice concomitant with the growth of the NCs. As an outcome, the NCs were formed with a Cu deficient or sub-stoichiometric composition.

**Fig. 1:**
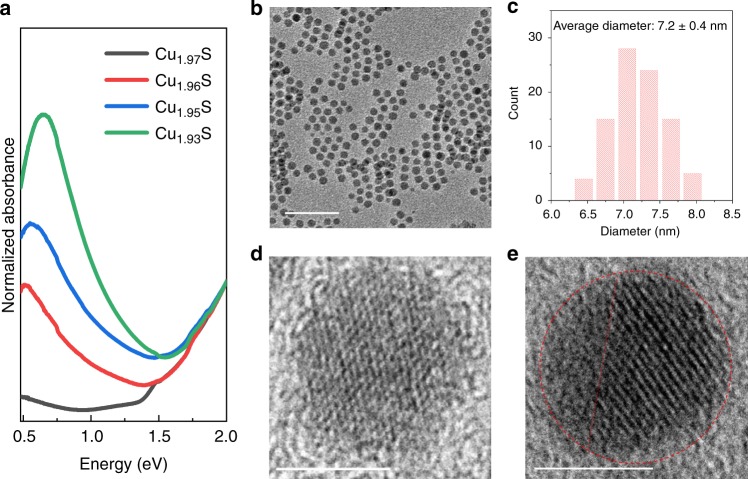
Characteristics of Cu_2−*x*_S NCs of sub-stoichiometric composition prepared by in-situ air exposure during hot-injection synthesis. **a** Vis–NIR extinction spectra (normalized to 1 at 2 eV) of colloids of Cu_2−*x*_S NCs of four different levels of sub-stoichiometry. Spectra are plotted in terms of energy in eV, which is approximated as 1240/(Wavelength in nm). **b** A wide-field TEM image (scale bar = 50 nm) of Cu_1.97_S NCs and **c** a histogram showing the NC diameter distribution determined from an analysis of the TEM image. The average diameter of the NCs is determined to be 7.2 nm with a standard deviation of 0.7 nm. Representative HRTEM image (scale bar = 5 nm) of a single NC of **d** Cu_1.97_S and **e** Cu_1.93_S. In **e** the NC boundary is marked by a dotted red circle and the domain boundary is marked by the dotted red line

The sub-stoichiometry (*x*) was determined by characterization of the localized surface plasmon resonance (LSPR) of the NCs^22,23^. Cu deficiencies contribute hole carriers to the valence band. These hole carriers result in the appearance of a LSPR band in the near-infrared region (NIR) of the extinction spectrum of the NC colloid (Fig. 1a). Whereas NCs synthesized in the absence of air exhibit only a weak absorption in the NIR region (Fig. 1a, black curve), NCs synthesized with in situ oxidation exhibit strong LSPR bands manifesting their Cu-deficient compositions. The larger the volume of air injected in the synthesis, the higher was the LSPR peak energy. It is well established that the peak energy, *ω*_*s*p_, is related to the hole carrier density, *N*_h_:

1 $$\omega _{{\mathrm{sp}}} = \sqrt {\frac{{N_{\mathrm{h}}e^2}}{{(1 + 2\varepsilon _{\mathrm{m}})\varepsilon _0m_{\mathrm{h}}}} - \gamma ^2}$$

where *e* represents the electronic charge, *ε*_0_ is the permittivity of free space, *ε*_m_ is the dielectric constant of the medium, *γ* represents the linewidth of the LSPR band, and *m*_h_ is the effective hole mass^22^. From the LSPR peak energy *ω*_sp_ and linewidth *γ* of 0.21 eV (ref. ^22^) and values of *m*_h_ = 0.8*m*_0_ (where *m*_0_ is the electron mass)^24^ and *ε*_*m*_ = 2.28 (for tetrachloroethylene)^22^, *N*_h_ (m^−3^) was estimated, which is a direct measure of the Cu defect density *δ* (mol cm^−3^).

2 $$\delta = 10^{ - 6}\frac{{N_{\mathrm{h}}}}{{N_{\mathrm{A}}}}$$

where *N*_A_ is Avogadro’s number. The level of sub-stoichiometry, *x*, is then obtained as:

3 $$x = \delta \frac{M}{\rho }$$

where *ρ* is the density of cuprous sulfide with a value of 5.6 g cm^−3^ and *M* is the formula weight for Cu_2_S with a value of 159.16 g mol^−1^. The resulting relationship between the stoichiometry and the LSPR peak frequency, *ω*_sp_, is best approximated as:

4 $$2 - x = 1.99336 - 0.15059\omega _{{\mathrm{sp}}}^2$$

We estimated the stoichiometry 2−*x* for the four different volumes of air-injection that we employed in our synthesis: 0 mL of air gave a composition of Cu_1.97_S, 0.01 mL gave Cu_1.96_S, 0.1 mL gave Cu_1.95_S, and 1 mL gave Cu_1.93_S. Thus, the Cu-deficiency level was controllable by the volume of air introduced. Although, the composition estimated from *ω*_sp_ using the above methodology is not expected to have absolute precision due to the approximations and uncertainties involved^22^, the estimate provides a reliable measure of relative Cu-deficiency level.

### Sub-stoichiometry-dependent internal structure of the NCs

**Fig. 2:**
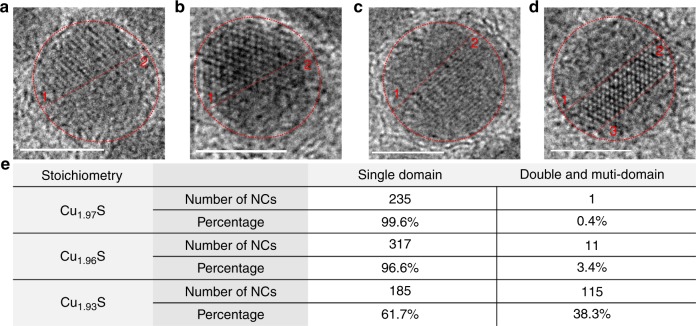
Internal domain structure. Representative examples of Cu_1.93_S NCs with multiple domains apparent in HRTEM imaging: NCs exhibiting **a**–**c** two domains and **d** three domains. In **a**–**d** the NC boundary is marked by a dotted red circle and domain boundaries are marked by dotted red lines. Domains are labeled as 1, 2, and 3. All scale bars are 5 nm in length. **e** Table of statistics of NCs exhibiting single- or multi-domain structures for three levels of sub-stoichiometry. The statistics were determined by analysis of NCs imaged by HRTEM

From transmission electron microscopy (TEM), the NCs were found to have an average diameter of 7 nm (Fig. 1b, c), which did not change much with the Cu-deficiency level. However, high-resolution TEM (HRTEM) images (Fig. 2 and Supplementary Figs. 1–3) showed a marked difference in the internal structure of the NCs of different Cu-deficiency level. As shown in Fig. 1d, e for representative NCs and summarized in a table in Fig. 2e for a population of over 200 NCs, Cu_1.97_S NCs are mainly single-domain, whereas a large fraction of Cu_1.93_S NCs show the presence of multiple domains. The representative NC shown in Fig. 1e exhibits two domains, one with distinct lattice fringes, while the other domain lacks lattice fringes. Additional examples of Cu_1.93_S NCs with multiple domains are shown in Fig. 2a–d and in Supplementary Fig. 1. While the presence of two domains is most prevalent for this stoichiometry, NCs with three or four domains are also occasionally observed (Fig. 2d and Supplementary Fig. 1). The prevalence of multiple domains follows a systematic trend as a function of the Cu-deficiency level. At a composition of Cu_1.97_S, only 1 of 236 NCs analyzed show multiple domains. At Cu_1.96_S, 11 of 328 NCs exhibit multiple domains. At Cu_1.93_S, 115 of 300 NCs exhibit two domains and a handful show three/four domains.

### Domains formed by twinning

**Fig. 3:**
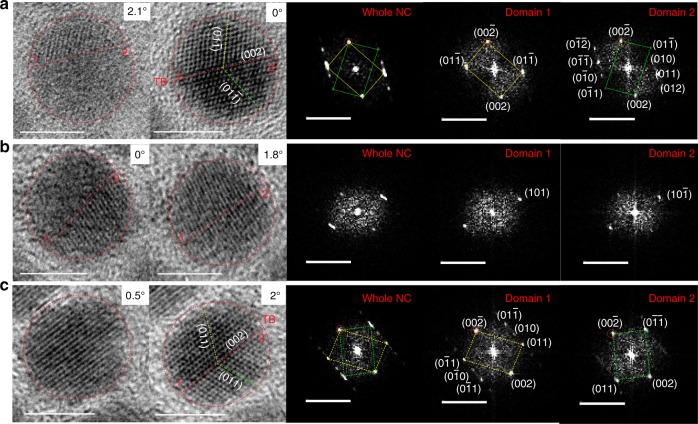
Identification of twinning. Twinned domains in Cu_1.93_S NCs verified by tilt-dependent HRTEM imaging of (**a–c**) three representative NCs. From left to right: image of NC without tilt, image of the same NC after a small tilt (about an axis located in the image plane oriented ~25° clockwise with respect to the vertical direction of the image plane), fast-Fourier transform (FFT) of the whole real-space image obtained under tilt, and select-area FFTs of the individual domains, labeled 1 and 2 in the real-space image. In the real-space images, the NC boundary is marked by a dotted red circle. The twin boundary (TB) is marked by a dotted red line and the reflection spot corresponding to the shared twin plane is marked by a dotted red circle in the FFTs. The crystallographic planes which have a mirror relationship across the twin boundary are marked by yellow (domain 1) and green (domain 2) dotted lines in the real-space image. Corresponding reciprocal lattice patterns for the two domains are indicated in the FFTs by parallelograms of the corresponding color. Several reciprocal lattice spots are assigned to specific planes of the high chalcocite phase and labeled by Miller indices. Scale bars in real-space images are 5 nm in length and those in FFTs are 5 nm^−1^

The multi-domain structure of the Cu_1.93_S NCs is an outcome of crystal twinning, as we found from a crystallographic analysis of the HRTEM images of NCs obtained at two different tilt angles. Results from three representative NCs are shown in Fig. 3. In the first example (Fig. 3a), the NC imaged without a tilt exhibits one domain with lattice fringes (Region 1) and another domain with none (Region 2). When imaged at a tilt of 2.1°, both domains show lattice fringes. The lattices of the two domains exhibit a twinning relationship.

Scanning transmission electron microscopy (STEM)-based three-dimensional 3D tomographic reconstruction would enable atomic-resolution visualization of the twinning in these NCs. However, our attempts at such characterization of the Cu_1.93_S NCs were unsuccessful, because the NCs exhibited low structural stability under the focused electron beam, making it infeasible to conduct atomic-resolution imaging at a range of orientations. Bright-field TEM imaging, which has been employed for the identification of twinning in nanostructures^2,5,7,8,12,13,25^, proved to be much more informative. Since lattice structures imaged by bright-field TEM can be complicated by the level of defocus, we ascertained the presence of twinning from the reciprocal lattice structure obtained by fast-Fourier transform (FFT) of the real-space HRTEM images (Fig. 3).

The FFT of the full NC contains a reciprocal lattice spot that is split into two, one contributed by each of the domains (Regions 1 and 2). Thus, the lattices of the two domains are rotated with respect to one another and exhibit a mirror image relationship. The twin boundary (TB) between the domains serves as a mirror reflection plane that is common to both domains. The twinning is of non-merohedral type, as indicated by the lack of complete overlap of the reciprocal lattice spot patterns of the two domains^26^.

Figure 3b and c present two other examples of such twinned domains within a NC. In the third example (Fig. 3c), the NC appears to be single-domain when imaged without tilt; but in the image acquired at a tilt of 1.8°, two domains with a twinning relationship are clearly observed. This example highlights that for some NCs, the zone-axis is perpendicular to the TB making it difficult to identify the twinned domains, unless the imaging plane is subject to a small tilt. As a result, the fraction of NCs reported in Fig. 2e to have multiple domains may be somewhat underestimated.

### Crystallographic structures of Cu_2−*x*_S NCs

**Fig. 4:**
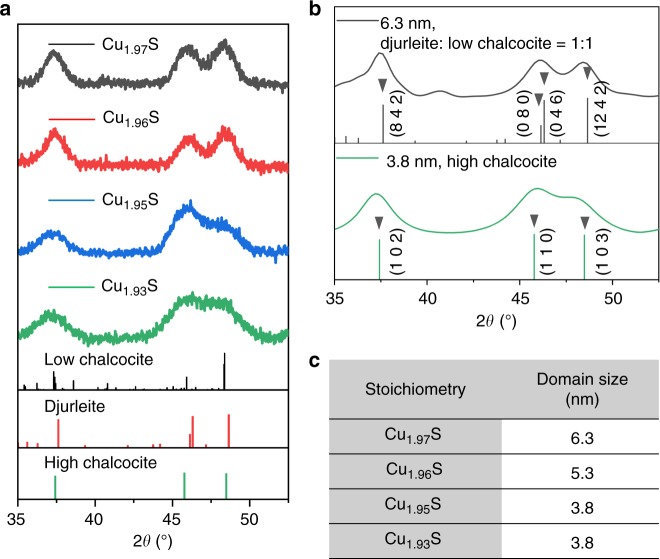
Sub-stoichiometry-dependent crystallographic structures of the NCs. **a** Experimental PXRD patterns of Cu_2−*x*_S NCs for four levels of sub-stoichiometry. Reference patterns are also shown for low chalcocite (JCPDS # 33–0490), djurleite (JCPDS # 23–0959) and high chalcocite (JCPDS # 26–1116) by stick patterns. **b** Simulated PXRD patterns of Cu_2−*x*_S NCs in a 1:1 mixture of djurleite: low chalcocite phase and a high chalcocite phase with key peaks assigned to specific Miller planes. For the input parameters for each simulated pattern, refer to Supplementary Tables 1–3. Reference patterns are also shown for djurleite (black sticks) and high chalcocite (green sticks). **c** NC domain size determined from Debye-Scherrer broadening (see Supplementary Table 4) of the 2*θ* ~37° reflection of the experimental PXRD pattern

Room temperature powder X-ray diffraction (PXRD) was employed to determine structures of the Cu_2−*x*_S NCs of all four stoichiometries (Fig. 4). The composition of Cu_1.97_S is intermediate between that of the fully stoichiometric (Cu_32_S_16_ or Cu_2_S) low chalcocite phase and the stable Cu-deficient (Cu_31_S_16_ or Cu_1.9375_S) djurleite phase. The PXRD pattern of Cu_1.97_S NCs is closely modeled by a simulated pattern of a 1:1 mixture of low chalcocite and djurleite phases (Fig. 4b) with a domain size of 6.3 nm, which was determined on the basis of Debye-Scherer broadening of the 2*θ* ~37° reflection (Fig. 4c). The domain size is close to the average NC diameter of 7.2 nm determined by TEM (Fig. 1c), which is consistent with the mostly single-domain nature of the Cu_1.97_S NCs.

Cu_1.93_S NCs have the stoichiometry of the djurleite phase^14^. The PXRD pattern of these NCs can be assigned to the djurleite phase^27^, but the reflection peaks appear at slightly smaller 2*θ* (Fig. 4a), yielding closer agreement with the high-temperature phase, which is defined by a hexagonal arrangement of S^2−^, a mobile Cu^+^ sub-lattice, and a 1.5 % larger unit cell volume than the djurleite phase, which has an immobile Cu^+^ sub-lattice^28^. The high temperature form of djurleite (which we refer to as high djurleite), is crystallographically indistinguishable from the high chalcocite phase despite the Cu-deficiency of the former. Due to the mobile, disordered Cu^+^ sub-lattice, the crystal arrangement is dominated by the S^2−^ sub-lattice.

Another key observation is the broadening of the PXRD peaks as the Cu-deficiency level increases from Cu_1.97_S to Cu_1.93_S (Fig. 4a). In fact, in the Cu_1.93_S NCs, the peaks at 2*θ* of ~46° and ~48° are fully overlapped. The larger peak broadening for the Cu_1.93_S NCs is indicative of smaller domains, as further verified by the domain size calculated from Debye-Scherrer broadening of the 2*θ* ~37° peak (Fig. 4c). The average domain size (3.8 nm) is appreciably smaller than the average NC diameter (7.2 nm), which is consistent with the multi-domain structure of the Cu_1.93_S NCs resulting from twinning. The average domain size is found to decrease with an increase in the Cu-deficiency level (Fig. 4c). This trend goes hand-in-hand with the observed increase in the prevalence of the multi-domain NCs with increasing *x* (Fig. 2e).

### Origin of twinning in Cu-deficient NCs

**Fig. 5:**
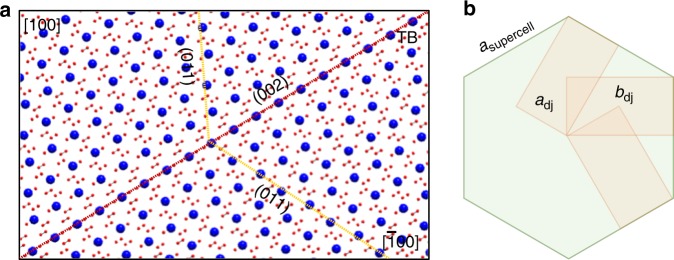
Origin of twinning. **a** Schematic of twinning in a high chalcocite lattice (S^2−^ shown in blue; Cu^+^ in red). The twin boundary (TB) is denoted by the dotted red line and key crystallographic planes are indicated by dotted yellow lines. **b** Symmetry relationship of a unit cell of near-orthorhombic djurleite (camel tetragon) to its supercell (green hexagon) of pseudo-hexagonal symmetry^41^. *a*_dj_ and *b*_dj_ are lattice constants of the djurleite unit cell, while *a*_supercell_ is the lattice constant of the supercell in the basal plane. The symmetry relationship is possible because *a*_dj_ ~√3 *b*_dj_

The Cu-deficient djurleite form has a monoclinic unit cell (*a*_dj_ *=* 26.90 Å, *b*_dj_ *=* 15.75 Å, *c*_dj_ *=* 13.57 Å, *β*_dj_ = 90.13°) which is nearly orthorhombic and has a special relationship between its lattice parameters: *a*_dj_ ~√3*b*_dj_^29^. As a result, multiple monoclinic unit cells can fit into a higher-symmetry supercell of pseudohexagonal structure, as illustrated in Fig. 5b. The twinning of the monoclinic structure is favored as a result of this symmetry relationship. At the high temperature at which the synthesis is performed, the NCs are formed with a high-temperature hexagonal arrangement of S^2−^. Cooling imposes a transition from this higher-symmetry hexagonal form to the lower symmetry monoclinic form. The lost symmetry element instead becomes a twin law: the monoclinic lattice formed is non-merohedrally twinned such that it adopts a hexagonal supercell^26^. This explains why NCs synthesized with the djurleite composition undergo twinning (Fig. 5a).

On the other hand, the stoichiometric low chalcocite unit cell (*a*_lc_ *=* 15.25 Å, *b*_lc_ *=* 11.88 Å, *c*_lc_ *=* 13.49 Å, *β*_lc_ = 116.35°) does not exhibit such a symmetry relationship^29^. As a result, NCs closer to low chalcocite in composition, do not favor twinning. The propensity of twinning increases as we go from a composition of Cu_1.97_S to Cu_1.93_S, i.e., as the djurleite composition is approached.

### Influence of twinning on phase transition characteristics

**Fig. 6:**
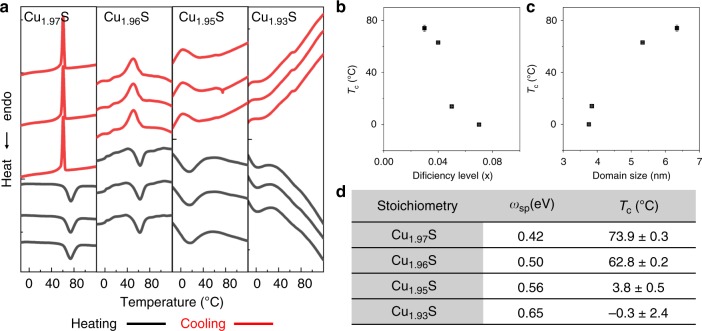
Effect of twinning on phase transition characteristics. **a** DSC thermograms of ca. 7 nm diameter Cu_2−*x*_S NCs of four levels of sub-stoichiometry. In each case, thermograms from three consecutive heating–cooling cycles performed on the same sample of NCs are shown, from which the reproducibility of the phase-transition peaks (dips) is evident. The thermograms are vertically offset for stacking. The peak temperature of the phase-transition in the heating sub-cycle was averaged across the three cycles to yield *T*_c_ and the standard deviation in *T*_c_. *T*_c_ plotted as a function of **b** the Cu-deficiency level, *x*, and **c** the domain size determined from PXRD. Error bars representing the standard deviation are shown for each data-point; but they are not resolvable in cases where the standard deviation is small. **d** Table of characteristics of the Cu_2-*x*_S NCs as a function of the deficiency level. Values of *ω*_sp_ correspond the LSPR peak maxima from Fig. 1a. The tabulated *T*_c_ values represent the average ± standard deviation, also plotted in **b**, **c**. The analysis described here was conducted for several batches of NCs and the variability in *ω*_sp_, stoichiometry, and *T*_c_ was determined (see Supplementary Table 5)

The twinned structure of the Cu_2−*x*_S NCs influences their properties, specifically their phase and phase transition characteristics, as revealed by differential scanning calorimetry (DSC) measurements. Figure 6a shows the DSC thermograms of Cu_2−*x*_S NCs of the four different Cu-deficiency levels. In each case, the endothermic peak observed in the heating sub-cycle of the thermogram signifies a phase transition from the low-temperature phase to the high-temperature superionic phase of Cu_2−*x*_S. The peak maximum is defined as the phase transition temperature (*T*_c_). Upon cooling, the phase transition is reversed as signified by the exothermic peak. The reproducibility of the transition peaks over multiple heating–cooling cycles signify the reversibility of the phase transition and the thermostability of the NCs over the course of the phase cycling.

For Cu_1.97_S NCs, the *T*_c_ is observed to be 74 °C, whereas in the bulk, the temperature for such a transition is ~104 °C (for chalcocite) and 97 °C (for djurleite)^14^. The lower *T*_c_ for the NCs is due to the nanoscale size effect, which is known to result in a depression in phase transition temperatures^30–32^. For the Cu_1.93_S NCs, which possess a twinned multi-domain structure (Fig. 2e), the *T*_c_ is 0 °C, which is 74 °C lower than that of the single-domain Cu_1.97_S NCs. In fact, there is systematic decrease in the *T*_c_ as *x* increases (Fig. 6b–d) and the average domain size decreases (Fig. 6c) due to the increased prevalence of twinned NCs. With increasing *x*, the phase transition peak also becomes broader, which is likely an outcome of heterogeneity in the domain structure and/or domain size across NCs. Thus, twinning results in a marked depression in the phase transition temperature of Cu_2-*x*_S. What is most noteworthy is that the Cu_1.93_S NCs are in a superionic phase at room temperature. This is also why the Cu_1.93_S NCs are found from room-temperature PXRD to be in the high-djurleite phase (Fig. 4a, b).

**Fig. 7:**
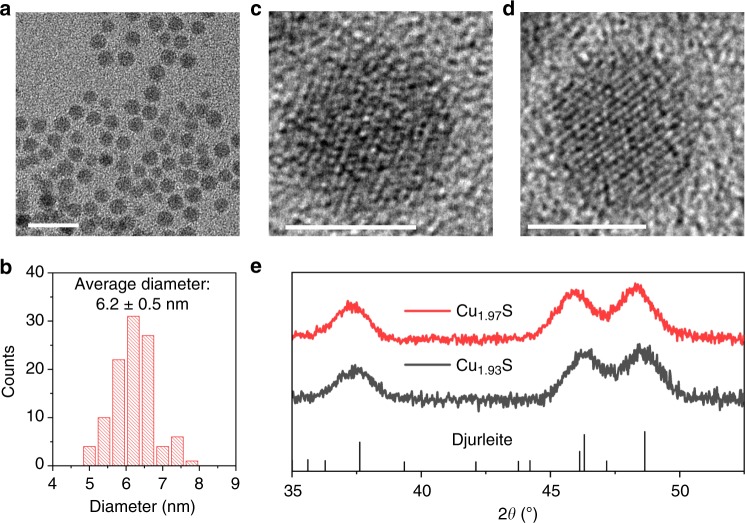
Structural characteristics of Cu_1.93_S NCs prepared by post-synthetic air oxidation. **a** Wide-field TEM image (scale bar = 20 nm) of Cu_1.93_S NCs synthesized by the hot-injection method and **b** histogram showing NC diameter distribution determined by ImageJ analysis of the TEM image in **a**. The average diameter of the NCs is determined to be 6.2 nm with a standard deviation of 0.5 nm. HRTEM image (scale bar = 5 nm) of a representative NC of **c** Cu_1.97_S and **d** Cu_1.93_S obtained from Cu_1.97_S by post-synthetic air exposure. **e** experimental PXRD patterns of Cu_1.97_S NCs and Cu_1.93_S NCs prepared by air oxidation of Cu_1.97_S NCs for 24 h. The reference pattern of djurleite phase (JCPDS # 23–0959) is shown by the stick plot. The sub-stoichiometry was estimated from the LSPR peak energy for the corresponding NCs (0.42 eV: Cu_1.97_S and 0.65 eV: Cu_1.93_S)

**Fig. 8:**
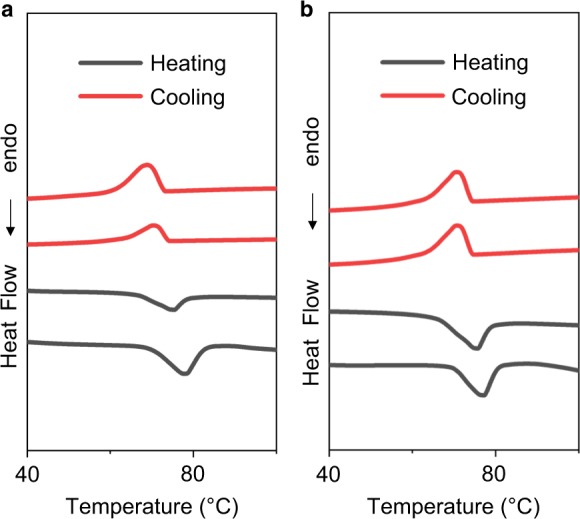
Phase-transition characteristics of Cu_1.93_S NCs prepared by post-synthetic air oxidation. DSC thermograms of **a** Cu_1.97_S NCs synthesized by hot-injection and **b** Cu_1.93_S NCs obtained from post-synthetic air exposure of Cu_1.97_S NCs for 12 h. In each case, thermograms from two heating–cooling cycles performed on the same sample of NCs are shown, from which the reproducibility of the phase-transition peaks (dips) is evident. The thermograms are vertically offset for stacking. The peak temperature of the phase-transition in the heating sub-cycle was averaged across the two cycles to yield *T*_c_. Note this study was performed on a different batch of NCs compared to the one in Fig. 7

It was necessary to examine if the depression in the *T*_c_ in Cu_1.93_S NCs is not simply the result of Cu deficiencies and is definitely an outcome of their twinned structure. Cu deficiencies in the Cu_2_S lattice can result in lattice compression. Compressive lattice strain has been demonstrated to aid the stabilization of superionic phases of selenides at lower temperatures than in the unstrained lattice^33,34^. A control study was performed with NCs with a Cu-deficient Cu_1.93_S composition but no twinned domains. In this study, Cu_1.97_S NCs synthesized with no air exposure, were subject to post-synthetic air-oxidation to form more Cu-deficient Cu_1.93_S NCs. The resulting Cu_1.93_S NCs have an average diameter of 6.2 nm and a single-domain structure similar to the as-synthesized Cu_1.97_S NCs (Fig. 7a–d and Supplementary Fig. 4). The average domain size is also unchanged upon post-synthetic oxidation, as manifested by the width of the PXRD peak at 2*θ* ~37° (Fig. 7e). In other words, post-synthetic oxidation did not result in twinning or multi-domain formation, unlike in-situ oxidation accomplished during hot-injection synthesis. Room-temperature PXRD shows that the Cu_1.93_S NCs are in the djurleite phase, and not the high-temperature superionic form (Fig. 7e). DSC characterization (Fig. 8) shows that as-synthesized Cu_1.97_S NCs and Cu_1.93_S NCs formed by post-synthetic oxidation have a similar *T*_c_ of 75 °C. These results indicate that a Cu-deficient composition alone, without twinning, does not lead to the *T*_c_ depression observed. Rather, twinning is responsible for the dramatic *T*_c_ depression and the room-temperature stabilization of the superionic phase in the Cu_1.93_S NCs synthesized by in-situ oxidation.

### Mechanistic origin of twinning-induced *T*_c_ depression

**Fig. 9:**
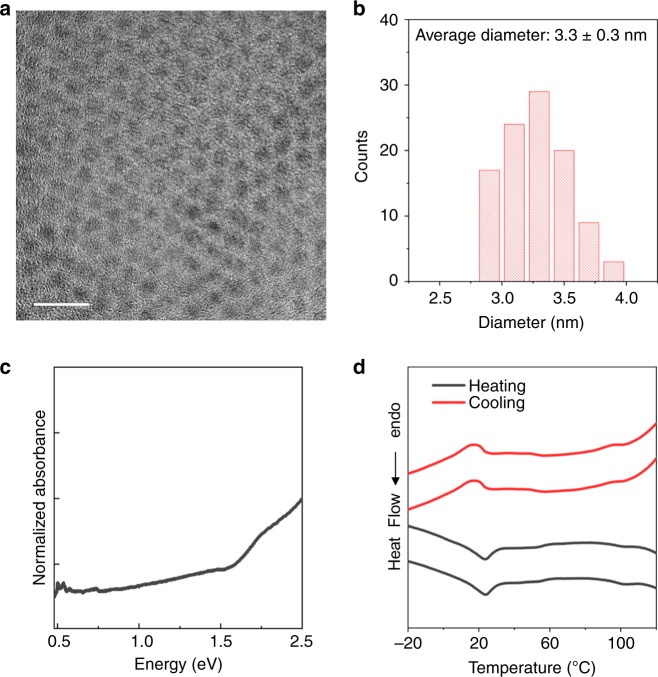
Characteristics of Cu_2−*x*_S NCs of a smaller size. **a** Representative TEM image (scale bar = 10 nm) of Cu_2−*x*_S NCs of a smaller diameter prepared with no air-injection and **b** histogram showing the NC diameter distribution determined by ImageJ analysis of the TEM image. The average diameter of the NCs is determined to be 3.3 nm with a standard deviation of 0.3 nm. **c** Vis–NIR extinction spectrum (normalized to 1 at 2 eV) of the Cu_2−*x*_S NCs of 3.3 nm average diameter. The NCs synthesized with no air-injection are expected to have a stoichiometry of Cu_1.97_S analogous to the case of ca. 7 nm diameter NCs, however the LSPR absorption of these smaller NCs was too weak to assign an *ω*_sp_. It is possible the NCs are nearly Cu_2_S in stoichiometry. **d** DSC thermograms of the these NCs. Thermograms from two heating–cooling cycles performed on the same sample of NCs are shown, from which the reproducibility of the phase-transition peaks (dips) is evident. The peak temperature of the phase-transition in the heating sub-cycle was averaged over the two cycles to yield a *T*_c_ of 24 °C with a standard deviation of 0.3 °C

The formation of smaller domains and additional domain boundaries within a NC by twinning is the likely cause of the substantial *T*_c_ depression. Such an effect of smaller domains is best demonstrated by a study of Cu_2−*x*_S NCs of a smaller diameter (Fig. 9a–c and Supplementary Fig. 5). NCs of ca. 7 nm diameter synthesized without air-injection show a *T*_c_, on average, of 75 °C (Supplementary Table 5); whereas NCs of a smaller diameter of ca. 3 nm synthesized without air-injection show a significantly lower *T*_c_ of 24 °C (Fig. 9d).

Previously, we had found that in small cuprous selenide nanoclusters, the superionic phase is observed at much lower temperatures than in the bulk solid^34^. While phenomenologically similar, the depression in the *T*_c_ seen here for the twinned Cu_2−*x*_S NCs is purported to be different in mechanistic origin. In cuprous selenide, the superionic phase has a compressed lattice as compared to the ordered phase^35,36^. As a consequence, compressive strain prevalent in small nanocrystals results in the stabilization of the superionic phase^33^. In contrast, in cuprous sulfide, the superionic high chalcocite phase has nearly the same lattice volume as that of the low chalcocite phase and a marginally larger lattice volume than the djurleite phase^37^. Compressive strain is, therefore, not likely to cause the *T*_c_ depression seen here.

Similar to the effect of a decreased NC size^31^, twinning results in an increase in the interfacial energy^38^, albeit without a change in the overall volume of the NC. This interfacial energy contributed by twin boundaries can lead to a depression in the Cu^+^ sub-lattice melting temperature (i.e., the *T*_c_) as explained below:

5 $$T_{\mathrm{c}} = \frac{{\Delta H}}{{\Delta S}}$$

where Δ*H* and Δ*S*, respectively, represent the specific enthalpy and entropy change involved in the transition from the low-temperature phase to the superionic high-temperature phase. For the bulk solid:

6 $$T_{\mathrm{c}}^{{\mathrm{bulk}}} = \frac{{\Delta H^{{\mathrm{bulk}}}}}{{\Delta S}}$$

But for a NC, the specific enthalpy of the low-temperature phase has an additional contribution from the non-negligible interfacial energy of the NC surface, therefore:

7 $$\Delta H = \Delta H^{{\mathrm{bulk}}} - \frac{{\zeta _{\mathrm{s}}.A_s}}{{\rho .V}}$$

where *ζ*_s_ is the surface energy (per unit area), *ρ* is the density, and *A*_s_ and *V* are, respectively, the surface area and volume of the NC. Furthermore, if the NC has internal twin boundaries, there is an addition interfacial energy contribution, therefore:

8 $$\Delta H = \Delta H^{{\mathrm{bulk}}} - \frac{{\zeta _{\mathrm{s}}.A_{\mathrm{s}}}}{{\rho .V}} - \frac{{\zeta _{{\mathrm{TB}}}.A_{{\mathrm{TB}}}}}{{\rho .V}}$$

where *ζ*_TB_ is the interfacial energy of twin boundaries (per unit area) and *A*_TB_ is the net area of the twin boundaries. Thus, for a NC with twin boundaries:

9 $$T_{\mathrm{c}}^{{\mathrm{TNC}}} = \frac{{\Delta H^{{\mathrm{bulk}}} - \frac{{\zeta _{\mathrm{s}}.A_{\mathrm{s}}}}{{\rho .V}} - \frac{{\zeta _{{\mathrm{TB}}}.A_{{\mathrm{TB}}}}}{{\rho .V}}}}{{\Delta S}} = T_{\mathrm{c}}^{{\mathrm{bulk}}}\left( {1 - \frac{{\zeta _{\mathrm{s}}.A_{\mathrm{s}}}}{{\rho .V}} - \frac{{\zeta _{{\mathrm{TB}}}.A_{{\mathrm{TB}}}}}{{\rho .V}}} \right)$$

which implies that the *T*_c_ of a NC with twin boundaries, (i.e., $T_{\mathrm{c}}^{{\mathrm{TNC}}}$) would be lower than that of the bulk crystal due to the combined effect of the NC surface and the twin boundaries. The relative depression in the *T*_c_ for a twinned NC with respect to the bulk value (i.e., $T_{\mathrm{c}}^{{\mathrm{bulk}}}$) is given as:

10 $$\frac{{\left( {T_{\mathrm{c}}^{{\mathrm{bulk}}} - T_{\mathrm{c}}^{{\mathrm{TNC}}}} \right)}}{{T_{\mathrm{c}}^{{\mathrm{bulk}}}}} = \left( {\frac{{\zeta _{\mathrm{s}}.A_{\mathrm{s}}}}{{\rho .V.\Delta H^{{\mathrm{bulk}}}}} + \frac{{\zeta _{{\mathrm{TB}}}.A_{{\mathrm{TB}}}}}{{\rho .V.\Delta H^{{\mathrm{bulk}}}}}} \right)$$

The first term on the right-hand side represents the depression caused by the nanoscale size. The smaller the NC size, the higher the surface area-to-volume ratio (*A*_s_/*V*) and the larger is this contribution. The second term on the right-hand side represents the additional depression caused by the presence of twin boundaries. Higher the density of twin boundaries (*A*_TB_/*V*), greater is this depression. This simple model thus explains the reduction in the *T*_c_ caused by the presence of twinning.

In summary, we have developed a method for the preparation of Cu_2−*x*_S NCs with a tunable internal domain structure. The method employs in situ oxidation during hot-injection synthesis for the growth of NCs with a defined Cu-deficiency level. By tuning of the Cu-deficiency level, NCs were synthesized with structures ranging from single-domain to multi-domain. The formation of multiple domains within Cu-deficient NCs occurs via crystal-twinning. It is found that the internal structure influences the ionic structure and properties of the NCs. NCs of ca. 7 nm diameter with twinned domains are stable in the superionic phase at temperatures as low as 0 °C; whereas for single-domain NCs, the transition to the superionic phase occurs only above 74 °C. The contribution of twin boundaries to the interfacial energy of the NCs is thought to cause the marked depression of the transition temperature. Not only do these findings open up the use of earth-abundant Cu_2−*x*_S as a room-temperature superionic conductor, but they also highlight, more generally, the utility of internal structural engineering for nanoscale materials design. The nanostructures developed here provide opportunities for investigating the influence of twinned interfaces on electronic and ionic transport^39^.

## Methods

### Chemicals

Copper (II) acetylacetonate (Cu(acac)_2_, Alfa Aesar, 98%), 1-octadecane (ODE, Sigma-Aldrich, 90%), 1-dodecanethiol (DDT, Sigma-Aldrich, > 98%), acetone (Fisher, 99.5%), hexane (Sigma-Aldrich, 98.5%), and tetrachloroethylene (Alfa Aesar, 99%).

### Synthesis of Cu_2−*x*_S NCs with in situ oxidation

A common synthesis procedure known from literature was modified to incorporate in situ oxidation^21^. In a typical synthesis, 0.1 g of Cu(acac)_2_ and 0.3 mL of DDT were mixed in a 25 mL three-neck, round-bottom glass flask, which was followed by the addition of 2.8 mL of ODE. The flask was then connected to a Schlenk line and subject to a vacuum for 0.5 h, after which the flask was brought to an Ar environment. To achieve a controlled degree of in-situ oxidation, a specific volume of air (0 mL, 0.01 mL, 0.1 mL, and 1.0 mL) was injected into the flask. Air was injected using a 1 mL syringe. For injection of 0.1 and 1 mL volumes, air was injected into the three-neck flask through a rubber septum capping one of the side-necks. For injection of a 0.01 mL volume, a gas mixture of Ar and air in a volumetric ratio of 9:1 was prepared in a glass vial. Then, 0.1 mL of this gas mixture was injected into the reaction flask using a 1 mL syringe. The solution was heated to 200 °Cunder stirring and allowed to react for 1.5 h. The reaction was stopped by removing the heater and allowing the flask to cool down to room temperature. The Cu_2−*x*_S NC colloid resulting from the reaction was washed with acetone and hexane and NCs were isolated by centrifugation (10,500 rpm for 10 min) for three times. The purified NCs were dispersed in 5.0 mL of hexane for further characterization. All post-synthetic processing of NCs and sample preparation was performed under inert gas (Ar or N_2_). The presence of air in the high-temperature synthesis resulted in the formation of NCs of a Cu-deficient composition by the oxidative removal of Cu from the Cu_2_S lattice.

The sub-stoichiometry (*x*) was determined from the peak energy of the LSPR band (*ω*_sp_) in the visible–near-infrared (vis–NIR) extinction spectrum measured for the NCs. Vis–NIR extinction spectra of the NCs were collected in a 1 cm pathlength NIR-transparent cuvette using a Shimadzu UV-3600 UV–vis–NIR spectrophotometer. The NCs were dispersed in tetrachloroethylene because this solvent has high transmission across the NIR region of interest. Typically, 0.2 mL of the as-synthesized NC colloid was mixed with 0.4 mL of acetone and centrifuged at 8000 rpm for 10 min. The precipitated NCs were redispersed in 2 mL of tetrachloroethylene for measurement of the vis–NIR extinction spectrum. From the *ω*_sp_, the sub-stoichiometry was found to be dictated by the volume of air introduced: (0 mL: Cu_1.97_S: 0.01 mL: Cu_1.96_S, 0.1 mL: Cu_1.95_S, and 1 mL: Cu_1.93_S). Further increase in the volume of air introduced or even conducting the hot-injection synthesis fully exposed to air did not yield NCs appreciably more sub-stochiometric than the Cu_1.93_S composition, which likely reflects the fact that the djurleite form with a composition in the vicinity of Cu_1.93_S represents a thermodynamically stable phase of Cu_2−*x*_S in air^40^.

The size of the NCs was tuned by adjusting the molar ratio of Cu(acac)_2_ to DDT. The reagent amounts listed above resulted in NCs of ca. 7 nm average diameter. NCs of a smaller ca. 3 nm average diameter were obtained by decreasing the reaction time to 45 min while keeping all other conditions the same. An alternative method for obtaining ca. 3 nm diameter NCs is to conduct the synthesis using 0.15 g of Cu(acac)_2_, 0.1 mL of DDT, and 3.8 mL of ODE while keeping the reaction time at 1.5 h.

### Post-synthetic oxidation of Cu_2−*x*_S NCs

The synthesized NC colloid was diluted a few-fold with hexane and exposed to ambient air at room temperature under stirring. Typically, vis–NIR extinction spectra of the colloid were collected at periodic intervals (0.5, 2, 12, and 24 h) to determine the Cu-deficiency resulting from the air oxidation. From the LSPR peak energy, the NCs appear to reach a sub-stoichiometry of Cu_1.93_S after 12 h of air oxidation; NCs subject to air oxidation for 12 or more hours were assigned a stoichiometry of Cu_1.93_S.

### Transmission electron microscopy

TEM images of the NCs were collected on a JEOL 2010F instrument operating at 200 kV. Unless noted otherwise, samples for TEM were prepared by drop-casting tens of microliters of the NC colloid onto a carbon-coated Formvar film on a 200-mesh Cu grid (Ted Pella, 01800-F). Images were analyzed for NC size determination using the software, Nano Measurer. Crystallographic analysis of TEM images was performed using Digital Micrograph and ImageJ. NC boundaries and internal domains were identified manually in ImageJ.

### Differential scanning calorimetry

Cu_2−*x*_S NCs of different size, Cu-deficiency level, and method of oxidation were characterized by DSC performed on a DSC Q20 V24.10 Build 122 or a TA Discovery 2500 instrument. For sample preparation, the NCs were isolated by subjecting the purified colloid to a non-solvent followed by centrifugation at 8000 rpm for 10 min. The precipitate was dried by application of vacuum. The resulting dried powder was transferred and compressed into a zero-T DSC aluminum pan. Thermograms were measured at a scan rate of 10 °C/min. Prior to the measurement, the sample was subject to a heating pre-cycle from −40 to 160 °Cfor desorbing any remaining ligands from the NC surfaces, which may otherwise contribute to complicating features in the thermogram. Multiple successive heating–cooling cycles were performed for testing the reproducibility of the measured thermogram, the reversibility of the phase transition, and the stability of the NCs over the course of the phase cycling.

### Powder X-ray diffraction

PXRD patterns of the NCs were collected on a Rigaku Miniflex 600 powder X-ray diffractometer operated at full power (40 kV, 15 mA) with Cu *K*_*α*_ radiation of a wavelength of 1.54 Å. Patterns were collected in reflection mode in the 2*θ* range of typically 20–80° using a step size of 0.02° with scans running for ~2 h. Typically, NCs were isolated from 500 μL of the colloid by centrifugation. The pellet was dispersed in 50 μL of hexane. The resulting colloid was drop-cast onto a zero-background quartz sample holder. Patterns were subject to manual baseline subtraction performed in Origin and appropriately scaled and vertically offset for presentation.

PXRD patterns were simulated using the PowderCell program, which performs a structure factor calculation using lattice parameters and atomic positions as input parameters. The input parameters for each simulated pattern are provided in Supplementary Tables 1–3. All simulated patterns include Debye-Scherrer broadening corresponding to a domain size estimated from experimental PXRD patterns (Supplementary Table 4). Simulated patterns were subject to manual baseline subtraction and were appropriately scaled and vertically offset for presentation.

## Supplementary information

Supplementary Information

## Data Availability

All raw images and source data are available from the authors upon reasonable request.
