# Supplementary material for: Room-temperature superionic-phase nanocrystals synthesized with a twinned lattice
Source: Nat Commun. 2019 Jul 23;10:3285. doi: 10.1038/s41467-019-11229-2 (PMC6650484; doi:10.1038/s41467-019-11229-2)
Supplement: Supplementary file 1 — Supplementary Information [file 41467_2019_11229_MOESM1_ESM.pdf]

Supplementary Information

Room-Temperature Superionic-Phase Nanocrystals Synthesized with a Twinned Lattice

Gong and Jain

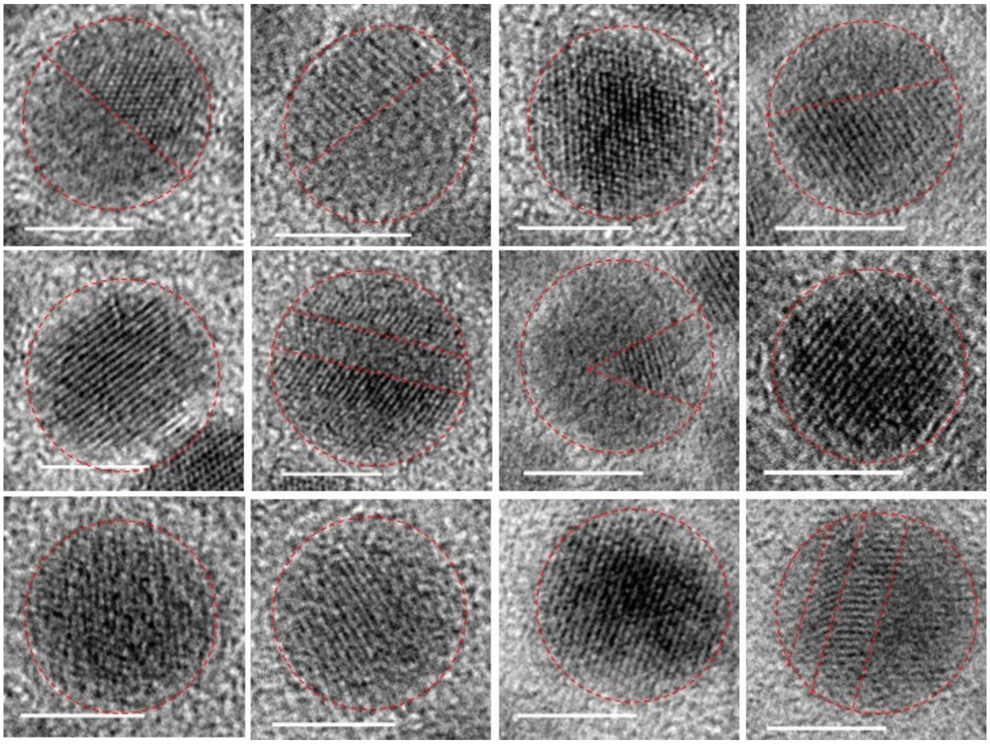

Supplementary Figure 1. High-resolution transmission electron microscopy (HRTEM) images of additional examples of individual $Cu_{1.93}S$ NCs of ca. 7 nm diameter. The boundaries of the NC are emphasized by the dotted red circle. For NCs with multiple domains, the domain boundaries are highlighted by dotted red lines. Each scale bar represents 5 nm.

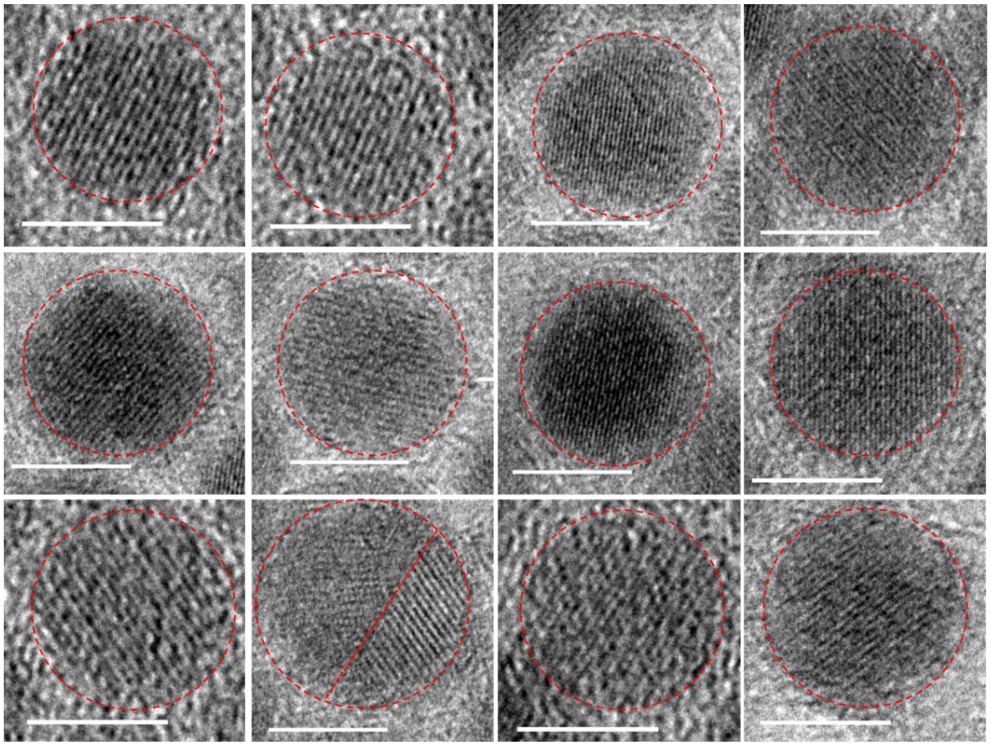

Supplementary Figure 2. HRTEM images of several examples of individual $Cu_{1.96}S$ NCs of ca. 7 nm diameter. The boundaries of the NC are emphasized by the dotted red circle. For the NC with multiple domains, the domain boundaries are highlighted by dotted red lines. Each scale bar represents 5 nm.

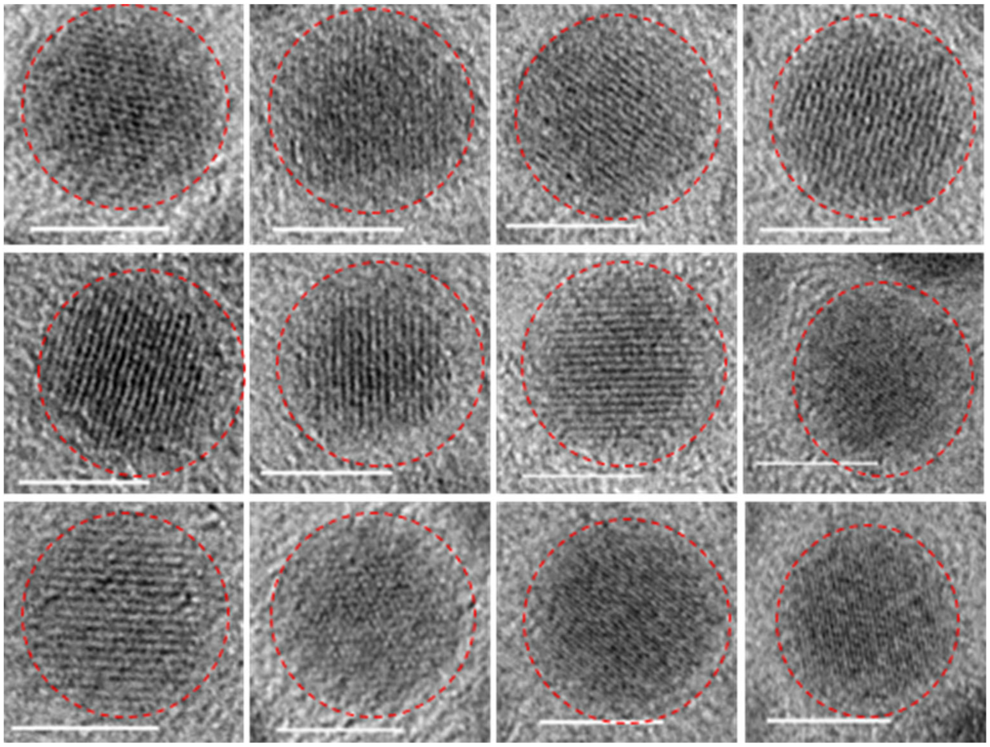

Supplementary Figure 3. HRTEM images of additional examples of individual $Cu_{1.97}S$ NCs of ca. 7 nm diameter. The boundaries of the NC are emphasized by the dotted red circle. None of the NCs in these examples exhibited multiple domains. Each scale bar represents 5 nm.

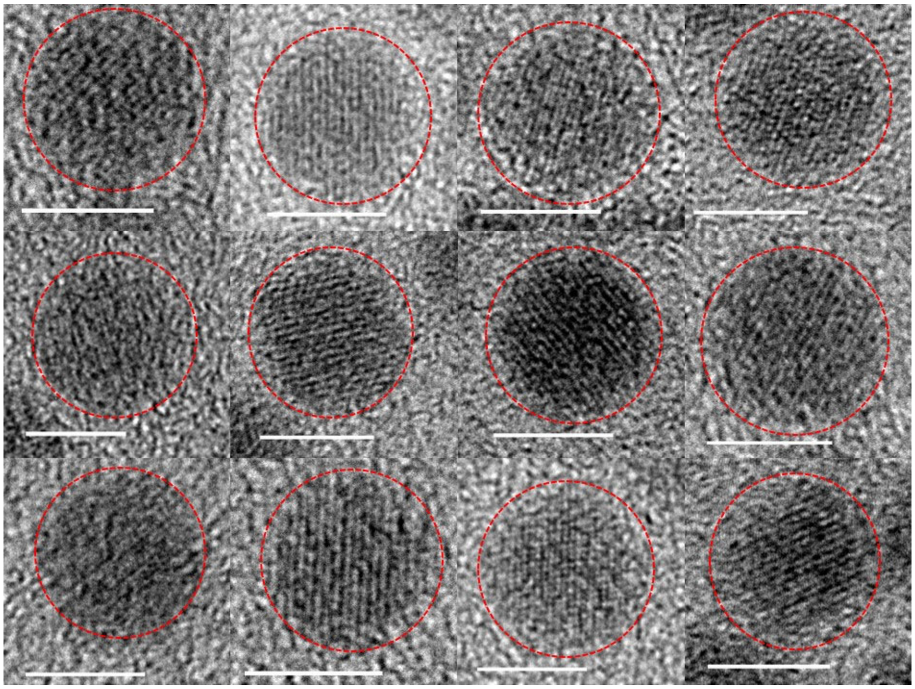

Supplementary Figure 4. HRTEM images of additional examples of individual $Cu_{1.93}S$ NCs prepared by post-synthetic air-exposure of $Cu_{1.97}S$ NCs of ca. 6 nm diameter. The boundaries of the NC are emphasized by the dotted red circle. Each scale bar represents 5 nm.

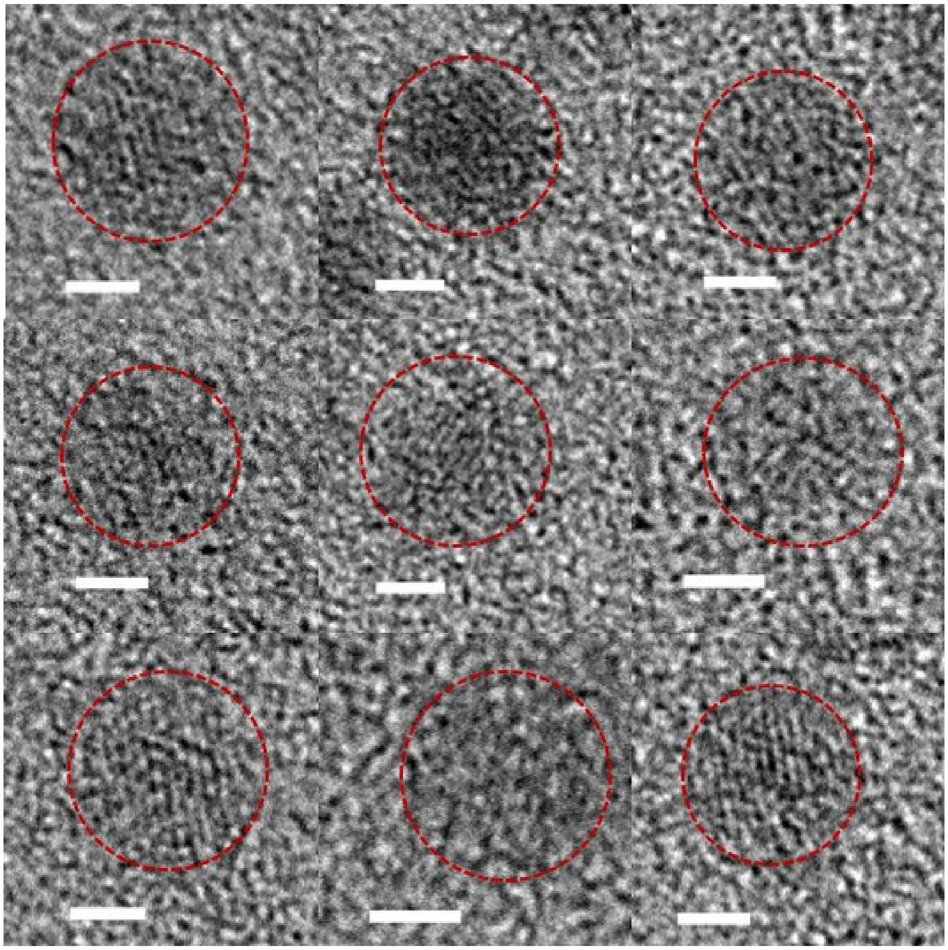

Supplementary Figure 5. HRTEM images of individual $Cu_{2-x}S$ NCs of ca. 3 nm diameter prepared by hot-injection without any air injection. The boundaries of the NC are emphasized by the dotted red circle. Each scale bar represents 2 nm. Only in this case, the HRTEM sample was prepared by drop-casting few microliters of a NC colloid onto an ultrathin carbon film on a lacey carbon support film on 300-mesh Au grid (Ted Pella, 01824G). The grid was then allowed to dry in a vacuum desiccator at 40 °C overnight to remove excess ligands or other organic contaminants.

**Supplementary Table 1. Crystallographic parameters[1] for simulated diffraction patterns in Figure 4b**					
**djurleite**	Space group: $P2_1/n$<br>a_{dj} = 26.897 Å b_{dj} = 15.745 Å c_{dj} = 13.565 Å<br>α_{dj} = 90 ° β_{dj} = 90.13 ° γ_{dj} = 90 °				
Element	Wycoff Pos.	*X*	*Y*	*Z*	Occ.
S	4 e	0.065	0.992	0.173	1
S	4 e	0.057	0.257	0.161	1
S	4 e	0.058	0.513	0.175	1
S	4 e	0.056	0.755	0.175	1
S	4 e	0.061	0.118	0.414	1
S	4 e	0.057	0.372	0.421	1
S	4 e	0.070	0.633	0.411	1
S	4 e	0.067	0.864	0.414	1
S	4 e	0.057	0.993	0.663	1
S	4 e	0.066	0.235	0.667	1
S	4 e	0.065	0.490	0.668	1
S	4 e	0.069	0.746	0.672	1
S	4 e	0.065	0.131	0.927	1
S	4 e	0.064	0.375	0.915	1
S	4 e	0.062	0.629	0.925	1
S	4 e	0.066	0.873	0.902	1
S	4 e	0.189	0.129	0.073	1
S	4 e	0.187	0.382	0.080	1
S	4 e	0.182	0.624	0.079	1
S	4 e	0.184	0.869	0.074	1
S	4 e	0.189	0.004	0.321	1
S	4 e	0.193	0.256	0.340	1
S	4 e	0.188	0.496	0.338	1
S	4 e	0.195	0.747	0.328	1
S	4 e	0.183	0.124	0.577	1
S	4 e	0.183	0.377	0.574	1
S	4 e	0.190	0.622	0.580	1
S	4 e	0.187	0.878	0.574	1
S	4 e	0.185	0.999	0.825	1
S	4 e	0.188	0.261	0.829	1
S	4 e	0.192	0.509	0.839	1
S	4 e	0.191	0.753	0.826	1

Continued…					
Element	Wycoff Pos.	*X*	*Y*	*Z*	Occ.
Cu	4 e	0.0585	0.4995	0.0013	1
Cu	4 e	0.0629	0.7546	0.0057	1
Cu	4 e	0.0658	0.1287	0.2463	1
Cu	4 e	0.0666	0.9891	0.495	1
Cu	4 e	0.0547	0.2432	0.500	1
Cu	4 e	0.0637	0.5088	0.4961	1
Cu	4 e	0.0674	0.113	0.7588	1
Cu	4 e	0.0618	0.3601	0.7429	1
Cu	4 e	0.0684	0.6153	0.755	1
Cu	4 e	0.1882	0.9989	0.9975	1
Cu	4 e	0.1932	0.2538	0.9947	1
Cu	4 e	0.1801	0.7454	0.9895	1
Cu	4 e	0.1886	0.1258	0.2391	1
Cu	4 e	0.1944	0.6213	0.2498	1
Cu	4 e	0.1901	0.8707	0.2424	1
Cu	4 e	0.1817	0.9986	0.4833	1
Cu	4 e	0.194	0.7508	0.4955	1
Cu	4 e	0.1816	0.1337	0.7545	1
Cu	4 e	0.189	0.3852	0.7412	1
Cu	4 e	0.1931	0.8773	0.7385	1
Cu	4 e	0.0161	0.1536	0.0656	1
Cu	4 e	0.0169	0.3899	0.1376	1
Cu	4 e	0.018	0.6341	0.1245	1
Cu	4 e	0.0148	0.877	0.125	1
Cu	4 e	0.0146	0.2896	0.3053	1
Cu	4 e	0.0164	0.4645	0.3179	1
Cu	4 e	0.0177	0.7394	0.3335	1
Cu	4 e	0.014	0.1048	0.5903	1
Cu	4 e	0.0112	0.388	0.5622	1
Cu	4 e	0.0176	0.8598	0.6328	1
Cu	4 e	0.0143	0.9668	0.8129	1
Cu	4 e	0.1082	0.0815	0.0721	1

Continued…					
Element	Wycoff Pos.	*X*	*Y*	*Z*	Occ.
Cu	4 e	0.1068	0.3423	0.0638	1
Cu	4 e	0.1032	0.9108	0.050	1
Cu	4 e	0.1081	0.5347	0.312	1
Cu	4 e	0.112	0.7677	0.3252	1
Cu	4 e	0.1086	0.9563	0.3169	1
Cu	4 e	0.1082	0.659	0.5622	1
Cu	4 e	0.1075	0.8336	0.5589	1
Cu	4 e	0.1049	0.0073	0.8886	1
Cu	4 e	0.1077	0.250	0.8788	1
Cu	4 e	0.1129	0.4712	0.8329	1
Cu	4 e	0.1091	0.774	0.8161	1
Cu	4 e	0.1392	0.4992	0.1277	1
Cu	4 e	0.1372	0.7337	0.1493	1
Cu	4 e	0.1392	0.1697	0.4233	1
Cu	4 e	0.1397	0.3639	0.4205	1
Cu	4 e	0.1404	0.0064	0.6588	1
Cu	4 e	0.1453	0.2554	0.610	1
Cu	4 e	0.1443	0.5029	0.6162	1
Cu	4 e	0.142	0.596	0.9293	1
Cu	4 e	0.2241	0.2886	0.1852	1
Cu	4 e	0.2303	0.462	0.1977	1
Cu	4 e	0.2346	0.1367	0.4227	1
Cu	4 e	0.2346	0.3904	0.4062	1
Cu	4 e	0.2308	0.5872	0.4358	1
Cu	4 e	0.2344	0.5411	0.6927	1
Cu	4 e	0.2325	0.7142	0.6811	1
Cu	4 e	0.2306	0.4097	0.9373	1
Cu	4 e	0.2373	0.6349	0.8738	1
Cu	4 e	0.2359	0.8561	0.9123	1
Cu	4 e	0.127	0.2612	0.2454	1

**Supplementary Table 2. Crystallographic parameters[2] for simulated diffraction patterns in Figure 4b**					
**low chalcocite**		Space group: $P2_1/c$ a_{lc} = 15.246 Å b_{lc} = 11.884 Å c_{lc} = 13.494 Å α_{lc} = 90 ° β_{lc} = 116.35 ° γ_{lc} = 90 °			
Element	Wycoff Pos.	*X*	*Y*	*Z*	Occ.
Cu	4 e	0.8647	0.2496	0.2923	1
Cu	4 e	0.6171	0.0733	0.6766	1
Cu	4 e	0.6100	0.0906	0.1673	1
Cu	4 e	0.3627	0.2392	0.0743	1
Cu	4 e	0.1283	0.0858	0.9452	1
Cu	4 e	0.1060	0.0789	0.4422	1
Cu	4 e	0.9340	0.1228	0.9916	1
Cu	4 e	0.9413	0.1413	0.5094	1
Cu	4 e	0.7617	0.2505	0.4112	1
Cu	4 e	0.4431	0.1488	0.9352	1
Cu	4 e	0.4259	0.1235	0.4390	1
Cu	4 e	0.2581	0.2355	0.8512	1
Cu	4 e	0.8212	0.0360	0.6834	1
Cu	4 e	0.7832	0.0623	0.1673	1
Cu	4 e	0.0264	0.2044	0.7726	1
Cu	4 e	0.5020	0.0800	0.2837	1
Cu	4 e	0.3030	0.0434	0.6232	1
Cu	4 e	0.3050	0.0431	0.1343	1
Cu	4 e	0.5251	0.2085	0.7543	1
Cu	4 e	0.9994	0.0854	0.2174	1
Cu	4 e	0.6229	0.1043	0.9537	1
Cu	4 e	0.7034	0.1939	0.5653	1
Cu	4 e	0.2028	0.2082	0.1405	1
Cu	4 e	0.1307	0.0963	0.6792	1
S	4 e	0.9570	0.0847	0.8420	1
S	4 e	0.9399	0.0774	0.3457	1
S	4 e	0.7924	0.0827	0.5068	1
S	4 e	0.7918	0.0830	0.0054	1
S	4 e	0.4495	0.0873	0.6140	1

**Continued**					
Element	Wycoff Pos.	*X*	*Y*	*Z*	Occ.
S	4 e	0.4444	0.0721	0.0948	1
S	4 e	0.3020	0.0776	0.7881	1
S	4 e	0.2834	0.0817	0.2865	1
S	4 e	0.6972	0.2482	0.7223	1
S	4 e	0.5479	0.2244	0.4168	1
S	4 e	0.1960	0.2384	0.4752	1
S	4 e	0.0475	0.2323	0.1338	1

Supplementary Table 3. Crystallographic parameters[3] for simulated diffraction patterns in Figure 4b					
**high chalcocite**		Space group: $P6_3/mmc$<br>a_{hc} = 3.959 Å b_{hc} = 3.959 Å c_{hc} = 6.784 Å<br>α_{hc} = 90 ° β_{hc} = 90 ° γ_{hc} = 120 °			
Element	Wycoff Pos.	*X*	*Y*	*Z*	Occ.
S	2 d	0.33333	0.66667	0.75	1
Cu	2 b	0	0	0.25	1
Cu	12 k	0.25730	0.51460	0.06610	1

Supplementary Table 4. Other simulation parameters for simulated diffraction patterns in Figure 4b			
**Pattern**	**2θ (°)**	**FWHM (°)**	**Peak function**
djurleite, 6.3 nm	37.35	1.38	Lorentz
low chalcocite, 6.3 nm	37.35	1.38	Lorentz
high chalcocite, 3.8 nm	37.00	2.32	Lorentz

The relationship between domain size and FWHM is based on the Debye-Scherrer expression according to which: domain size in nm = 0.94*λ_X/(cosθ*FWHM in radian) where λ_X is Cu K_α X-ray radiation wavelength of 0.154 nm.

**Supplementary Table 5. Characteristics of NCs from multiple batches of synthesis conducted as per the procedure of ca. 7 nm diameter NCs.**				
Volume of air (mL)	Number of batches	ω_{sp} (eV)	Stoichiometry	T_c (°C)
0	3	0.42 ± 0.01	1.967 ± 0.000	75.1 ± 1.0
0.01	4	0.48 ± 0.02	1.958 ± 0.003	60.2 ± 3.6
0.1	10	0.53 ± 0.02	1.951 ± 0.004	10.6 ± 5.2
1	3	0.67 ± 0.01	1.926 ± 0.003	0.4 ± 0.4

For each batch of NCs, ω_{sp}, stoichiometry, and T_c were determined as described in the main text. The value of each parameter averaged over all batches is listed along with a ± range that corresponds to a standard deviation. For the 0 mL case, for one of the batches, the LSPR band was too weak to determine ω_{sp} or stoichiometry, so this batch was not included in the determination of the average and standard deviation in ω_{sp} or stoichiometry. The phase transition peak in DSC thermograms was weak in the case of NCs synthesized with 1 mL of air injection: for two of the batches, the peak could not be resolved reliably, so these batches were not included in the determination of the average in T_c and the standard deviation was determined from multiple cycles of thermograms measured from one batch of NCs.

Supplementary References

1. Evans Jr., H. T. Djurleite ($Cu_{1.94}S$) and low chalcocite (Cu_2S): New crystal structure studies. *Science* **203**, 356–358 (1979).

2. Evans Jr., H. T. Crystal structure of low chalcocite. *Nat. Phys. Sci.***232**, 69–70, (1971).

3. Cava, R., Reidinger, F. & Wuensch, B. J.. Mobile ion distribution and anharmonic thermal motion in fast ion conducting Cu_2S. *Solid State Ion.* **5**, 501–504 (1981).
